# Survey and Associated Risk Factors for the Presence of Ruminant Pestiviruses in Domestic Ovine and Caprine Populations from Kazakhstan

**DOI:** 10.3390/v17050676

**Published:** 2025-05-06

**Authors:** Andrey V. Zhigailov, Yuliya V. Perfilyeva, Angelina A. Malysheva, Alena S. Cherusheva, Zhanna A. Berdygulova, Dinara A. Naizabayeva, Karina R. Ivanova, Saltanat A. Kuatbekova, Zhaniya M. Dosmagambet, Anzhelika V. Lushova, Sofiya A. Kan, Artyom V. Kuligin, Akerke O. Bissenbay, Moldir M. Kuatbek, Akzhigit S. Mashzhan, Nurshat Abdolla, Anna S. Nizkorodova, Elina R. Maltseva, Aralbek S. Rsaliyev, Yergali O. Abduraimov, Ainur A. Zhaksylykova, Aida M. Abdybekova, Seidigapbar M. Mamadaliyev, Yuriy A. Skiba, Yekaterina O. Ostapchuk

**Affiliations:** 1 Almaty Branch of the National Center for Biotechnology, National Holding “QazBioPharm”, 14 Zhahanger St., Almaty 050054, Kazakhstan; andrzhig@gmail.com (A.V.Z.); malysheva.angelina.alx@gmail.com (A.A.M.); cherusheva_a@mail.ru (A.S.C.); berdygulova@gmail.com (Z.A.B.); dinara.naizabaeva@gmail.com (D.A.N.); karina.rusl.2022@gmail.com (K.R.I.); kuatbek9205@gmail.com (S.A.K.); zhaniya.dosmagambet@gmail.com (Z.M.D.); anzhelika.lushova@gmail.com (A.V.L.); kan.soofiya@gmail.com (S.A.K.); kuligin.artyoom@gmail.com (A.V.K.); akerke.bissenbay@gmail.com (A.O.B.); moldirkuatbek698@gmail.com (M.M.K.); aj.akzhigit@gmail.com (A.S.M.); nurshata@gmail.com (N.A.); mysh.mysher@gmail.com (A.S.N.); maltsevaer@gmail.com (E.R.M.); mamadaliyev.s@bk.ru (S.M.M.); yuriy.skiba@gmail.com (Y.A.S.); katyostapchuk@gmail.com (Y.O.O.); 2 National Holding “QazBioPharm”, 13/1 Walikhanov St., Astana 010000, Kazakhstan; a.rsaliyev@qbp-holding.kz (A.S.R.); e.abduraimov@qbp-holding.kz (Y.O.A.); 3 Kazakh Scientific Research Veterinary Institute, National Holding “QazBioPharm”, 223 Raiymbek Avenue, Almaty 050016, Kazakhstan; ainusik_jan_91@mail.ru (A.A.Z.); aida_abdybekova@mail.ru (A.M.A.)

**Keywords:** bovine viral diarrhea, border disease, BVDV, BDV, small ruminants, *Pestivirus*, risk analysis, Kazakhstan

## Abstract

Pestiviruses, particularly bovine viral diarrhea virus (BVDV), cause significant economic losses worldwide. While cattle are the primary hosts for BVDV, sheep and goats can also be affected. This nationwide survey aimed to assess the prevalence, genetic characteristics, and risk factors associated with pestiviruses in sheep and goats in Kazakhstan. A one-off cross-sectional study was conducted to estimate the prevalence of pestiviruses in sheep and goats across 58 districts in 17 oblasts of Kazakhstan. A total of 2028 animals were examined using antibody ELISA, and RT-qPCR was performed on 2056 samples. Logistic regression models were used to identify potential risk factors linked to pestiviral infection. The overall prevalence of pestiviral infection in small ruminants was estimated to be 53.7% by ELISA and 2.5% by RT-qPCR. Regression analysis revealed that age, farm type, and geographic location were risk factors for pestiviral infections in small ruminants in Kazakhstan. Partial sequence analysis of the 5′-untranslated region confirmed the presence of BVDV2. Phylogenetic analysis revealed two distinct clusters of Kazakhstani BVDV2 strains, which were significantly different from known BVDV2 genotypes. No other ruminant pestiviruses were identified. The results highlight the importance of integrating small ruminants into BVDV infection control strategies to mitigate risks to livestock.

## 1. Introduction

Bovine viral diarrhea virus (BVDV) is one of the most important viral pathogens in cattle. It causes a range of disease syndromes in cattle, including enteric and respiratory disease, reproductive disorders, immunosuppression, and reduced growth rates. Infection with BVDV results in significant economic losses throughout the world [1]. Cattle are considered the natural host of BVDV. However, many other animal species are susceptible to infection with BVDV, including sheep, goats, buffalo, yaks, camelids, pigs, and wild ruminants, although they usually undergo subclinical infection [2]. These species of animals may serve as a reservoir capable of sustaining the pathogen in the environment and pose a major, poorly understood threat to the livestock industry [3].

As with other pestiviruses, BVDV is capable of crossing the placental barrier of pregnant animals and infecting the fetus [4]. BVDV infection can induce immunotolerance in utero, leading to lifelong persistent infection (PI) [5]. The PI animals are primarily responsible for the spread and maintenance of the virus in cattle populations [6]. Although BVDV vaccines are widely used as part of the BVDV control strategy, vaccination alone may be insufficient to prevent BVDV infection in large herds [7]. Effective control and eventual eradication of BVDV requires a comprehensive biosecurity strategy, vaccination, identification of BVDV reservoirs, and elimination of PI animals from infected herds [8]. Herds of sheep and goats should be considered an important potential reservoir of BVDV infection, as they can come into contact with cattle herds during free grazing [9]. This especially applies to countries with developed sheep and goat farming, such as Kazakhstan.

According to the International Committee on Taxonomy of Viruses [10], the BVDV belongs to the *Pestivirus* genus, family *Flaviviridae*, and is divided into two distinct species, *Pestivirus bovis* (BVDV1) and *Pestivirus tauri* (BVDV2). Based on the phylogenetic analyses, BVDV1 is currently separated into at least 21 subgenotypes (BVDV1a to BVDV1u), and BVDV2 into four subgenotypes (BVDV2a to BVDV2d) [11,12]. The symptom severities and virulence differences among BVDV1 and BVDV2 strains have been reported [13,14]. Differences in immune response to different BVDV subgenotypes were also presented [15,16,17]. These findings highlight the significance of genetic characterization of BVDV strains for disease prevention and vaccination programs.

Cross-reactivity between BVDV and some other pestiviruses has been reported to be a potential impediment in surveillance and diagnosis [18,19]. *Pestivirus ovis*, also known as border disease virus (BDV), primarily infects sheep and goats and causes a variety of clinical manifestations, including congenital disorders, abortion, stillbirths, tremors, abnormal body conformation, low birth weight, hairy fleece, and immunosuppression [20,21,22]. *Pestivirus brazilense*, also known as HoBi-like pestivirus (HoBiPeV), also occurs in cattle and small ruminants and causes clinical signs that are similar to those of BVDV infection [23,24]. The 5′-untranslated region (5′UTR) sequences have been primarily used for the genotyping and classification of pestiviruses, including BVDV [25,26]. This locus has also been most commonly used for phylogenetic analyses of BVDV isolates [17]. Consequently, we chose this locus for the phylogenetic analysis of the identified pestiviruses in this study.

Kazakhstan is a country located in the center of Eurasia, with a large territory mainly occupied by deserts and semi-deserts. Sheep farming and cattle breeding are the most important areas of animal husbandry in the country. The populations of sheep/goats and cattle in Kazakhstan reach 19,947,000 and 8,064,000, respectively (Supplementary Table S1) [27]. Until 2019, the territory of Kazakhstan was considered free from BVDV [28], but starting from 2019, mass outbreaks of BVDV infection began occurring in Kazakhstan [29]. In 2021–2023, outbreaks covered the entire territory of the country [29]. A state vaccination system for cattle against BVDV has been launched in high-risk regions. Although the state vaccination program annually involves only about 1.7% of the total cattle population (Supplementary Table S1), the owners of large and medium-sized enterprises have started to privately vaccinate their cattle. Approximately thirty vaccines for the immunization of cattle against BVD—all of which are inactivated—are approved for use within the Eurasian Economic Union. Kazakh farmers have access to all of these vaccines to immunize their cattle [30].

In 2021–2022, our research group conducted a one-off cross-sectional study to estimate the occurrence and risk factors of BVDV in cattle in Kazakhstan. According to that study, the rates of cattle vaccination against BVDV reached 65.2% [31]. We also detected BVDV2 in forest flies (*Hippobosca equine*) [32]. However, to date, no epidemiological survey of BVDV has been conducted on small ruminants in Kazakhstan. The prevalence and molecular epidemiology of BDV and HoBiPeV in Kazakhstan, to the best of our knowledge, also remain unexplored [22,29,33]. This work is the first epidemiological study estimating the pestivirus seroprevalence and RT-qPCR positive rate in sheep and goat flocks in Kazakhstan. Here, we also report the genetic characterization of BVDV2 first detected in Kazakhstani sheep.

The results contribute to our understanding of the importance of integrating small ruminants into BVDV infection research and control strategies to mitigate risks to livestock and wildlife.

## 2. Materials and Methods

### 2.1. Ethics Statement

The protocol of this study was approved by the local ethics committee of the National Center for Biotechnology, Astana, Kazakhstan (Approval #4, 03 December 2021 and Approval #3, 12 July 2023). Sampling was performed in accordance with ethical guidelines for animal research.

### 2.2. Study Area and Design

With a territory of 2,724,900 square kilometers, Kazakhstan is the largest landlocked country in the world. It is located in the heart of Eurasia between 45° and 87° E longitude, 40° and 55° N latitude, and shares borders with Russia, China, Kyrgyzstan, Uzbekistan, and Turkmenistan. More than three-quarters of the country is either semi-desert or desert. Sheep are the dominant livestock, with a population exceeding 20,210,671 [27]. The highest density of sheep and goats is in the country’s southern region. A large proportion of sheep and goats (52.7%) are housed in backyard farms [27].

A current cross-sectional study was conducted as part of a nationwide survey to examine the prevalence of the main viral pathogens of sheep and goats in the country. Permission for sampling was obtained from the Committee for Veterinary Control and Surveillance of the Ministry of Agriculture of Kazakhstan. Conducted from November 2022 to December 2024, the survey covered 58 districts across all 17 oblasts of Kazakhstan.

Veterinary centers in the oblasts were notified about the planned study, and designated specialists visited these centers to gather lists of all registered farms where sheep and goats are raised. Farms were randomly selected from these lists and contacted in advance. Visits were only conducted if the owner of the farm was available and agreed to participate. All animals on the participating farms were eligible for sampling. The minimum sample size was determined according to the following formula [34]:

n = Z^2^ P_exp_ (1 − P_exp_)/d^2^, (1)

where n = required minimum sample size; P_exp_ = expected prevalence; d = desired absolute precision; Z = normal distribution of the corresponding to the alpha value (1.96 for a 95% confidence level).

Based on an expected infection prevalence of 50% (commonly used when the actual prevalence is unknown), a 95% confidence level, and a maximum allowable error of 5%, the minimum sample size of 384 animals was determined.

The obtained sample size was further adjusted for design effects (DE) to accommodate the clustering of observations [35]:

DE = 1 + ρ (b − 1),(2)

where ρ = the intra-cluster correlation coefficient for BVDV infection; b = the expected average number of animals per cluster sampled (number of sheep or goats sampled per herd).

The sheep or goat herd here was considered a cluster. An average cluster size of 17 animals was used. The intra-cluster correlation coefficient for BVDV infection was reported as 0.23 [36]. Consequently, the adjusted minimum sample size was estimated to be 1797 animals.

### 2.3. Sample Collection

A total of 2462 sheep and 52 goats were sampled across 58 districts in 17 oblasts of Kazakhstan from November 2022 to December 2024. Out of 148 sampled flocks, six included animals of both species, 138 included sheep, and four flocks included only goats. Animals were restrained and handled gently to minimize stress, avoid animal injury, and limit movement during sample collection. Blood samples were collected from the jugular vein into 5 mL Vacutainer tubes containing K_2_EDTA or clot activator. The serum was separated from the clot before the samples were frozen. Nasal and rectal swabs were collected using cotton swabs and were immediately placed into transport tubes. The samples were transported in a mobile freezer at −20 °C to the laboratory for further analysis.

During the sampling, data were collected through interviews with farmers using a semi-structured questionnaire. The recorded information included the species, age, breed, sex, and physiological condition of the animals, as well as farm size and the geographical coordinates of the sampling locations. Only animals with recorded data on age, sex, and species were included in the further analysis.

### 2.4. Enzyme-Linked Immunosorbent Assay (ELISA)

Before conducting the ELISA, we assessed the hemolysis status of serum samples using an Elx808 ELISA reader (BioTek Instruments, Inc., Winooski, VT, USA), as described by Chatterjee et al. [37]. Serum samples exhibiting a low hemolysis index were then tested for antibodies against the highly conserved non-structural p80-p125 protein of BVDV/BDV using an ID Screen^®^ BVD p80 Antibody Competition Kit (IDVET, Grabels, France), according to the manufacturer’s protocol. This competitive ELISA kit is suitable for various species, including ovine and caprine species. The specificity and sensitivity of this competitive ELISA are 100% and 100%, respectively, according to the manufacturer.

### 2.5. RNA Isolation

To increase the likelihood of detecting pestiviruses in small ruminants, nasal and rectal swabs from the same animal were pooled during RNA extraction. RNA was extracted from pooled nasal and rectal swabs by selective precipitation using the MAGNO-sorb DNA/RNA extraction reagent kit (Form 4) (Amplisens, Moscow, Russia) on the automated Automag-96 station (Hangzhou Allsheng Instruments Co., Hangzhou, China). Extracted RNA was stored at −80 °C until analysis.

### 2.6. Pestivirus RNA Detection and Typing

We used a method validated by Hoffman et al. [38] to detect pestiviruses that can infect sheep and goats, specifically BVDV1, BVDV2, BDV, and HoBiPeV. This approach utilizes universal primers and a TaqMan probe for the ‘PanPesti’ RT-qPCR assay. Reactions were conducted in a single tube with 25 µL reaction volumes containing primers PestiV-189-qF, PestiV-396-qR, and the probe PestiV-235-qPr (Table 1), targeting the conserved 5′-untranslated region (5′-UTR) of pestiviruses [38]. The probe was produced and labeled with the fluorescent reporter FAM at the 5′-end and the quencher BHQ-1 at the 3′-end (Metabion International, Planegg, Germany). The RT-qPCR was performed using the MMLV Taq M master mix Lyo (Alkor Bio, Saint Petersburg, Russia) according to the manufacturer’s protocol.

Briefly, for a single well, 2 µL Rnase-free water (Biolabmix, Novosibirsk, Russia), 12.5 µL 2× reaction mix, 2 µL specific FAM-labeled primer–probe mix (10 pmol/µL specific primers + 1.25 pmol/µL probe) and 10 µL RNA template were added, followed by the real-time RT-qPCR on the CFX96 (BioRad, Fremont, CA, USA) instrument. The following temperature profile was used: 50 °C for 10 min (RT-step), 94 °C for 2 min, followed by 42 cycles at 94 °C for 30 s, 57 °C for 45 s and 68 °C for 45 s. Each run included both negative and positive controls to ensure the validity of the results. Identical temperature profiles were used for all real-time RT-qPCR runs, and fluorescence values were recorded during the annealing step. Threshold lines were determined at 10% of the positive amplification control’s amplification plateau. A cycle threshold (Ct) value of 40 was used as a cutoff.

Samples testing positive in the RT-qPCR were then analyzed using conventional semi-nested RT-PCR with species-specific primers for pestivirus typing, followed by Sanger sequencing. The cDNA was synthesized from the RNA using a reverse transcriptase M-MLV kit (New England BioLabs, Ipswich, MA, USA) with random hexamer primers (ThermoFisher Scientific, Waltham, MA, USA), according to the manufacturer’s instructions. Each reaction mixture contained 10 µL of sample RNA, 1 µL random hexamer primers, and 9 µL of reverse transcription solution. Temperature conditions were as follows: 25 °C for 10 min; 42 °C for 1 h; 85 °C for 10 min.

For the detection of BVDV1 and BVDV2, semi-nested RT-PCR was performed with the outer primers BVDV-101-F/PestiV-394-R [41], and the inner primers BVDV-105-nF/PestiV-394-R, yielding a 289-bp amplification product [42] (Table 1). To detect BDV RNA, the initial RT-PCR run utilized the outer primers BDV-136-F and PestiV-394-R (Table 1). In the second PCR, we employed the primers BDV-136-F and BDV-360-R, which amplified a 224-bp PCR product [43] (Table 1). The RNA of HoBiPeV was detected using a semi-nested RT-PCR with the outer primers HoBi-134-F/PestiV-394-R and the inner primers HoBi-134-F/HoBi-326-nR, which amplified a 192-bp fragment [44,45].

Amplification was carried out using Hot Start Taq DNA Polymerase (New England BioLabs, Ipswich, MA, USA) according to the manufacturer’s instructions. Each 20 µL reaction included 2.0 µL of the reverse transcription reaction product. Thermal cycling conditions consisted of a 95 °C pre-denaturation step for 5 min, followed by 40 PCR cycles of 94 °C for 25 s, 56–58 °C (annealing temperatures for each primer pair are indicated in Table 1) for 25 s, and 72 °C for 1 min, concluding with a 5-min incubation at 72 °C. PCR products were analyzed using 1.5% agarose gel electrophoresis and visualized under UV light. Only samples displaying distinct bands on the gel and with confirmatory DNA sequencing results were considered positive.

### 2.7. Sequencing and Phylogenetic Analysis

The PCR products of the expected size, after the purification with QIAquick gel extraction kit (Qiagen, Germantown, MD, USA), were directly sequenced using a BigDye Terminator v3.1 Cycle Sequencing Kit (Applied Biosystems, Carlsbad, CA, USA) and analyzed using a 24-capillary ABI 3500xl Genetic Analyzer (Applied Biosystems, Foster City, CA, USA).

The Basic GenBank Local Alignment Search Tool (BLAST) program (release 264.0) was used to compare the resulting nucleotide sequences with those deposited in the NCBI GenBank database [50] and to calculate the statistical significance of matches. Multiple sequence alignment was performed using the MUSCLE algorithm. The phylogenetic relationships among the analyzed isolates were established using maximum-likelihood algorithms and a model with the lowest Bayesian Information Criterion score. The Molecular Evolutionary Genetics Analysis (MEGA) X software ver. 10.1.8 (The Pennsylvania State University, State College, PA, USA) [51] was used for phylogenetic analysis. The bootstrap method with 1000 replicates was used to evaluate the reliability of the tree’s topologies [52].

The sequences reported in this work are available in the GenBank database [50] under the accession numbers PV138170–PV138183.

### 2.8. Statistical Analysis and Data Source

Regression analysis was performed using EpiInfo version 7.2.2.2 (CDC, Atlanta, GA, USA). The apparent prevalence of infection at the animal level was calculated by determining the proportion of positive animals among all those tested. Herd prevalence was defined as the proportion of herds containing at least one positive animal. Odds ratios (OR) with 95% confidence intervals (CI) were calculated to assess the strength of associations. The chi-square (χ^2^) test was used to detect statistically significant differences between groups. Differences were considered statistically significant at *p* < 0.05.

The screening of potential risk factors associated with sheep and goat seropositivity to ruminant pestiviruses (BVDV1, BVDV2, BDV and HoBiPeV) was primarily conducted using an unadjusted random-effects binary logistic regression model. The outcome variable was the apparent seroprevalence at the animal level. All independent variables were subsequently analyzed using an adjusted random-effects multivariate logistic regression model. The predictive model was fitted as follows [35]:

P = 1/(1+ e^−(α + ΣβiXi)^), (3)

where P is the probability of pestivirus infection; α is the y-intercept (constant); β_i_ is the regression coefficient for an independent variable X_i_; X_i_ is an epidemiological factor used to characterize each of the districts; *e* is the base of the natural logarithm (2.71828).

Predictors were selected based on biological relevance and exploratory data analysis. We investigated various factors, including the species tested (sheep and goats), the age of the test subjects (under 1 year old, 1–2 years old, and over 2 years old), sex (female and male), herd size (fewer than 100 heads, 100–500 heads, and more than 500 heads), and the type of property (personal subsidiary households, peasant farms, or limited liability companies and state enterprises). For the predictive model, we examined economic and geographic factors associated with individual pestivirus seropositivity in sheep and goats. These factors included the geographic region of the country, the density of small ruminants, the density of cattle, the density of wild ruminants, livestock importation from 2022 to 2024, the density of automobile routes, the proportion of sheep and goats in backyard farms, the shared border with countries where pestiviral infections are endemic, and the proximity to sites of previous BVDV outbreaks.

Data on the density of cattle, goats, and sheep, as well as farm sizes in the districts, were retrieved from the report of the Statistical Agency of the Republic of Kazakhstan [27]. Information on the prevalence of ungulate species was obtained from maps created by Afanasiev [53]. Data on the populations of various wild ruminant species susceptible to ruminant pestiviruses are available on the website of the Statistical Agency of the Republic of Kazakhstan [54]. We used data on the density of major automobile routes in Kazakhstan, estimated in ArcGIS by Meijer et al. [55]. Information on BVDV outbreaks and imported livestock was retrieved from the Official website of the State Inspection Committee in the Agro–Industrial Complex of the Ministry of Agriculture of the Republic of Kazakhstan [56].

## 3. Results

### 3.1. Sampling

**Figure 1:**
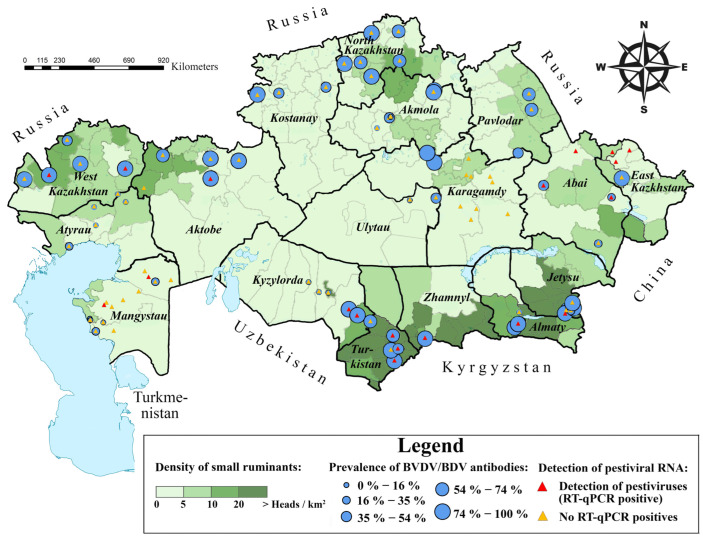
Sampling sites prevalence of BVDV/BDV antibodies per localities and settlements where RT-qPCR-positive animals were identified. The gradient of the green background reflects the density of the sheep and goat population. Blue circles indicate settlements included in serological analysis. The size of the blue circles represents BVDV/BDV seroprevalence levels within each settlement. Red triangles represent the locations where pestivirus shedding was confirmed through RT-qPCR or sequencing. Orange triangles represent settlements with no RT-qPCR-positive animals. The map was created using the geographic information system ArcMap software ver. 10.5.1 (Esri Inc., Redlands, CA, USA). The number of cattle per district was obtained from the official website of the Bureau of National Statistics of the Republic of Kazakhstan [27].

Overall, samples from 2514 animals from 148 herds were at our disposition (Figure 1 and Table 2). The median herd size for both sheep and goats was 151 animals, with a range of 15 to 3900. On average, 17.0 animals were sampled per herd, with a sampling range of 1 to 30 animals. The average age of the sampled animals was 3.32 ± 0.07 years (ranging from 2 months to 14 years). Females predominated in the sample (82.0%; 1820/2220; 95% CI: 80.3–83.5); gender information was not recorded for 165 animals. Most of the samples were obtained from crossbred sheep (1754 animals), with the remaining animals belonging to the following breeds: 300 Kazakh coarse-wool sheep, 140 Ordabasy, 124 Kazakh fine-wool sheep, 124 Kazakh fat-tailed coarse-wool sheep, and 20 Edilbay. All 52 goats sampled were crossbred. None of the sampled animals exhibited specific clinical symptoms of pestiviral infection, and no owners reported vaccinating their livestock against any pestiviral agents. For further analysis, only animals with recorded information on age and sex were selected.

### 3.2. Seroprevalence of Pestiviruses in Sheep and Goats and Risk Analysis

Of the 2514 samples collected, 129 were rejected due to the absence of important information about the animals, and 357 serum samples were unsuitable for further antibody ELISA due to a high degree of hemolysis. Therefore, only samples from 2028 animals (1980 sheep and 48 goats) were analyzed by antibody ELISA (Table 2). The serological analysis revealed an overall seroprevalence of 53.7% (1088/2028; 95% CI: 51.5–55.8) at the animal level and a herd-level seroprevalence of 86.7% (78/90; 95% CI: 77.9–92.9) (Table 2). Seroprevalence within herds ranged from 0% to 100%. Seropositive animals were detected in all 17 oblasts (Figure 1 and Table 2). A map displaying sampling sites and the spatial distribution of seropositive and RT-qPCR-positive animals, created using ArcMap software ver. 10.5.1 (Esri Inc., Redlands, CA, USA), is shown in Figure 1.

To assess the seroprevalence and the risk variables associated with the seroprevalence for pestiviruses in sheep and goats in Kazakhstan, we analyzed the results of a serological investigation using both univariate and multivariate logistic regression models. Univariate associations between BVDV/BDV seropositivity in animals and the risk variables at the individual animal level are presented in Table 3.

There were no significant (*p* > 0.05) differences in BVDV/BDV seroprevalence between sexes and species (sheep and goats) (Table 3). The seroprevalence for pestiviruses was found to be significantly higher in animals over two years of age than in young animals (Table 3). According to this study, the seroprevalence for pestiviruses was higher in sheep and goats from peasant farms (OR = 8.2; 95% CI: 6.6–10.3; *p* < 0.0001) compared to those from settlements with other property forms. Larger herd size was also associated with pestiviral infection (Table 3). We found statistically significant geographic differences in seroprevalence for pestiviruses in sheep and goats, ranging from 16.1% in Atyrau oblast (95% CI: 10.6–23.8) to 88.0% (95% CI: 68.3–83.3) in Zhambyl oblast (Table 2). The prevalence of antibodies to BVDV in sheep and goats was not statistically different in the western, central, and eastern regions of Kazakhstan; however, it was significantly higher in the southern (*p* = 0.0003) and northern (*p* < 0.0001) regions of Kazakhstan (Table 3).

All the identified variables considered potential risk factors for seroprevalence for pestiviruses, specifically: ‘age over 2 years’, ‘peasant farms’, ‘large herd size’, and ‘northern or southern region of Kazakhstan’, were included in a multivariate logistic regression model following univariate analysis. All four factors were found to be significantly associated with pestivirus seropositivity in sheep and goats (Table 4).

We further attempted to identify economic and geographic factors related to pestivirus seropositivity at the district level (Table 5). Univariate analysis suggested that the likelihood of BVDV/BVD introduction into a district was associated with several risk factors, ranked as follows: ‘shared border with countries where pestiviral infections are endemic’ > ‘high density of main automobile routes’ > ‘high density of small ruminants’ > ‘high density of cattle’ > ‘animal importation’ > ‘high proportion of backyard husbandry’ (Table 5). Interestingly, a high density of wild ruminants was found to be a protective factor. The category ‘proximity to sites of previous BVDV outbreaks’ showed no significant association with the outcome (Table 5).

All seven variables that demonstrated statistically significant associations, both positive and negative, with animal-level seroprevalence for pestiviruses were further analyzed using a multivariate model. Of these, five variables remained significantly associated with pestivirus seropositivity (Table 6). To calculate the probability of the outcome (*p*) for each district in the country, we used the coefficients for the variables obtained from the multivariate model, as outlined in Table 6.

**Figure 2:**
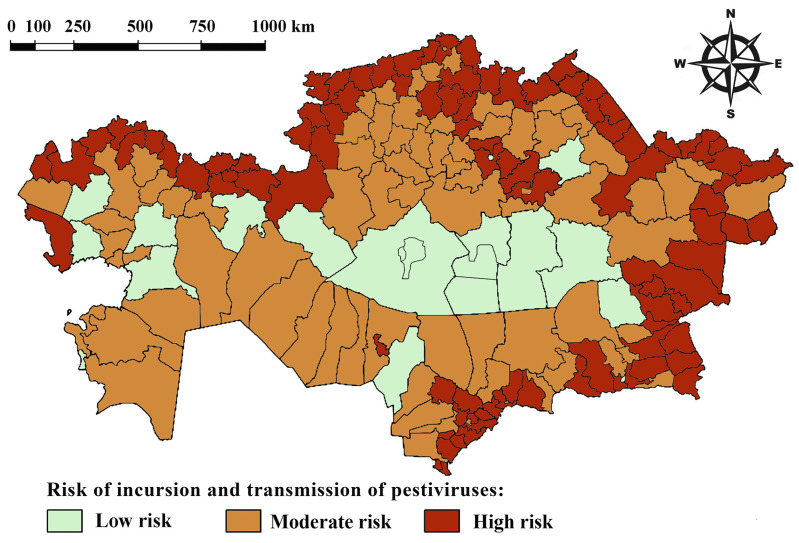
Risk map for pestiviral infections.

After processing the obtained data, we ranked all districts in the Republic of Kazakhstan based on their risk of pestiviral infection in sheep and goats. A risk map for the emergence of pestiviral infections was created (Figure 2). A district was classified as high risk if the probability (P) was greater than or equal to 0.8 and as moderate risk if the probability was between 0.6 and 0.8 (0.6 < P ≤ 0.8).

### 3.3. Molecular Detection and Genetic Characterization of Pestiviruses

RNA was extracted from biological specimens, including combined nasal and rectal swabs, collected from a total of 2230 animals (2187 sheep and 43 goats). Samples from 174 animals were excluded due to poor RNA extraction quality. As a result, 2056 samples were analyzed further by RT-qPCR. Of these samples, 2.5% (52/2056; 95% CI: 1.9–3.3) tested positive for RT-qPCR (Table 2). Among these RT-qPCR-positive animals, 25 were seropositive, and 11 were seronegative; ELISA was not conducted for the remaining 16 animals. The RT-qPCR-positive animals were identified in 10 of 17 oblasts (Figure 1).

To analyze and characterize the identified pestiviruses, all RT-qPCR-positive RNA samples were then subjected to a conventional semi-nested RT-PCR with species-specific primers targeting the conserved 5′UTR locus. Of the samples tested, 47 (90.4%) produced PCR products of the expected size (289 bp) using BVDV-specific primers (Table 2). However, no specific amplicons were generated with the BDV-specific and HoBiPeV-specific primers.

**Figure 3:**
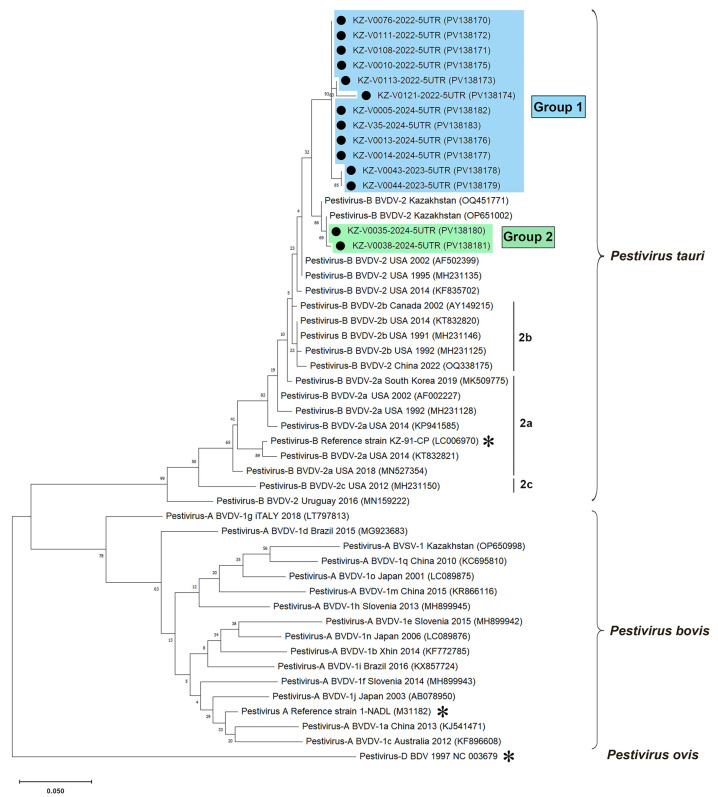
Phylogenetic analysis based on partial 5′UTR sequences (291–295 nt) of pestiviruses. A maximum-likelihood phylogenetic tree was constructed in MEGA X from alignments of fourteen partial 5′UTR sequences generated in this study and 34 database sequences using Kimura’s two-parameter method (K2+G model) [57]. The tree is drawn to scale, with the branch lengths representing the number of substitutions per site (the scale bar is present at the bottom of the phylogenetic tree). The percentage of replicated trees in which the associated taxa were clustered together in the bootstrap test (1000 replicates) is indicated at the nodes. The GenBank accession numbers are shown in parentheses. The Kazakhstani BVDV2 isolates determined in this study (marked with black circles (●)) are shown in blue or green rectangles, depending on their grouping. The asterisk is the reference genomes of BVDV1 [46] (GenBank: M31182), BVDV2 [47] (GenBank: LC006970), BDV [49] (GenBank reference sequence: NC_003679). The Pestivirus D strain was used as an outgroup.

To determine whether the detected strains were BVDV1 or BVDV2, the amplified fragments of the 5′UTR were directly sequenced, and sequencing was successful in 22 out of 47 PCR-positive samples. Nucleotide comparisons in BLAST revealed that all amplicons belonged to the BVDV2 species. We deposited 14 nucleotide sequences, which had good full-length amplicon sequences, in the GenBank database [50] for further use in phylogenetic analysis. Phylogenetic trees were constructed to illustrate the relationships between the identified BVDV2 genetic variants and other known strains (Figure 3).

Phylogenetic analysis of partial 5′UTR sequences of BVDV2 from Kazakhstan revealed two major groups, with a low mean nucleotide intragroup divergence reaching 1.0%. The overall mean genetic divergence (*p*-distance) between the two groups was found to be 4.2%. Most of the BVDV2 strains identified in this study (GenBank accession no. PV138170–PV138179, PV138182, PV138183) clustered into one clade, designated as Group 1. Representatives of this group were found in the Turkistan, Mangistau, Abai, East Kazakhstan, and Zhambyl oblasts of Kazakhstan. Pestiviruses belonging to Group 2 (GenBank accession no. PV138180 and PV138181), which were identified in the Aktobe oblast, clustered into a distinct clade alongside BVDV2 strains (GenBank accession no. PV138180, PV138181) that we found in cattle in the same oblast in 2021 [31].

Representatives of both Kazakhstani BVDV2 groups exhibited relatively low sequence similarity with strains of the BVDV2a and BVDV2b genotypes, averaging 97.2% and 96.8% for BVDV2a, and 97.4% and 96.6% for BVDV2b, respectively. Consequently, both groups of Kazakhstani BVDV2 strains could potentially be classified as separate BVDV2 genotypes. However, this hypothesis cannot be verified solely based on the analysis of 5′ UTR sequences.

## 4. Discussion

During our serological and molecular survey of BVDV in cattle [31], we observed that owners of large and medium-sized cattle farms vaccinate their cows against BVDV. However, in some cases, reliable information regarding the animals’ vaccination status was lacking. The situation is complicated by the market availability of combination vaccines containing BVDV. Unlike cattle, sheep and goats in Kazakhstan are not vaccinated against pestiviruses, making them suitable subjects for seromonitoring studies of BVDV. While the potential spread of BDV in sheep and goat populations—where they are the primary hosts—could complicate the interpretation of our results, we did not detect BDV RNA in the small ruminant populations of Kazakhstan.

The estimated animal-level prevalence of antibodies to pestiviruses in sheep and goats (53.7%) observed in this study was comparable to that in unvaccinated cattle in Kazakhstan (48.6%), as established during the 2020–2022 survey [31]. Seropositive small ruminants were found in all five regions of the country. The pestivirus seroprevalence found in this study is significantly higher than reported in similar studies of small ruminants across Asia and Europe. For example, in Korea, an individual seroprevalence of 1.49% and a herd seroprevalence of 11.67% were reported in black goats [58]. In India and the Tibetan Plateau, China, seroprevalence rates of 32.9% and 36.7% were observed in sheep, respectively [59]. In Switzerland, 16.3% of sheep tested positive for antibodies to pestiviruses [60]. These findings indicate a relatively high level of pestivirus exposure among small ruminants in Kazakhstan.

Further molecular screening of small ruminants revealed the presence of BVDV genetic material. RT-PCR-positive animals were identified in the western, southern, and eastern regions of Kazakhstan. The results obtained indicate that BVDV from sheep and goats should be considered an important source of BVD infection for domestic cattle. Partial sequencing of the 5′ UTR of BVDV strains has revealed the presence of at least two genetic variants of BVDV2 across the country. Additionally, at least two different genotypes of BVDV1 have also been identified within the cattle population [31]. This suggests that the infection likely entered the country multiple times from different sources. Although we did not detect either HoBiPeV or BDV in our study, we cannot exclude their circulation in sheep and goat populations in Kazakhstan.

The highest seroprevalence for pestiviruses in sheep and goats was observed in both the northern and southern regions of Kazakhstan. This may be observed due to the high density of cattle, sheep, and goats, as well as the high rates of breeding livestock importation from abroad to these regions [27]. To understand the factors contributing to this regional disparity, we analyzed multiple potential risk factors for pestiviral infection in sheep and goats. The obtained results can be used to adjust the state’s strategy for BVDV control. We found that pestiviral seroprevalence in several oblasts of Kazakhstan (e.g., Abai, Atyrau, and Ulytau oblasts) was significantly lower than the national average (Table 2, Figure 1). Many herds in these regions contained no seropositive animals. To prevent the further spread of the infection to non-endemic areas, it is crucial to implement effective infection control measures promptly. Studies have described geographic differences in pestivirus seroprevalence, including those conducted in China [61,62,63,64] and the Russian Federation [65], which share a border with Kazakhstan.

Several studies [66,67,68,69] support the finding that adult animals demonstrate higher seropositivity for BVDV and BDV compared to younger animals. This suggests that older animals are more likely to have been exposed to the virus over time. We found that larger herds were associated with higher odds of pestiviral infection. This is well aligned with the data from other researchers [70,71].

In this study, peasant farms were found to be riskier than personal subsidiary households, limited liability companies, and state enterprises. This finding is not surprising, as peasant farms in Kazakhstan typically raise significantly larger livestock compared to individual enterprises and, like personal entrepreneurs, primarily engage in extensive livestock farming. In peasant farms, livestock are usually kept free-range, which allows them to come into contact with animals from other farms. While the average number of sheep and goats in limited liability companies and state enterprises is often comparable to, or even higher than, that of peasant farms, these enterprises generally use intensive livestock farming practices. They conduct regular veterinary inspections and carefully implement preventive measures to reduce the risk of livestock infections.

Interestingly, the variable ‘density of susceptible wild ungulates’ emerged as a statistically significant protective factor against BVDV/BDV seropositivity. One possible explanation for this finding is that wild ungulates tend to inhabit areas with low levels of livestock farming and other economic activities. To explore this further, we examined the ‘high density of wild ruminants’ variable alongside potential risk factors related to the intensity of human economic activity. These risk factors included the density of cattle, the density of small ruminants, and high road density. We assessed collinearity among these variables using Pearson’s correlation coefficient. In each case, the correlation coefficient was negative but relatively weak, indicating the following values: −0.26 for ‘small ruminant density’, −0.17 for ‘cattle density’, and −0.11 for ‘high road density’. Perhaps the result depended on a combination of several factors.

It is somewhat surprising that the parameter ‘proximity to the sites of previous BVD outbreaks’ was not statistically linked to the level of pestivirus seropositivity. This can be attributed to the fact that, over the past five years, BVDV infection has spread rapidly throughout nearly the entire country. As a result, the initial locations of outbreaks are no longer significant for the ongoing spread of the infection.

The most likely primary source of BVD virus (BVDV) infection for sheep populations in Kazakhstan is infected cattle. This conclusion is supported by our findings, which showed that the *P. tauri* isolates we identified in cows in 2021 [31] (GenBank: OQ451770—OQ451772) exhibited over 98.5% homology in their partial 5′ UTR sequences with Group 2 of Kazakhstani BVDV2 strains (GenBank: PV138180, PV138181) identified in sheep in this study (Figure 3). We assume that BVDV was introduced into the country through imported infected livestock. In Kazakhstan, sheep are rarely imported, while goats account for a very small percentage of imported livestock, less than 0.5% [27]. The majority of imported livestock consists of cattle, making up 60–70% of the total [27]. Previous reports indicate that BVDV1 strains are predominant in Europe and Asia, including countries like Russia [70] and China [63], which border Kazakhstan. In contrast, BVDV2 strains are mainly found in the USA, Canada, and South America [12]. Interestingly, the most commonly detected pestivirus in Kazakhstan is *P. tauri*, suggesting that the virus may have entered the country from the Americas. Significantly, the first major outbreaks of the disease in Kazakhstan began at the end of 2018 [29]. Around the same time, European countries such as Denmark, Germany, and Hungary became the leading exporters of breeding cattle to Kazakhstan, whereas live cattle were primarily imported from the USA and Canada before 2018 [27].

There is an urgent need to develop a national strategy for infection control and eradication. Various BVD control programs are being implemented across Europe and the USA [72]. Several European countries have successfully carried out national eradication campaigns for BVDV [72]. Kazakhstan needs to select a strategy for BVDV control; however, this strategy may be inadequate if the interactions between sheep, goats, and cattle are not considered. The results obtained in this study can assist in the development and optimization of such a unified state control system against BVDV infection.

Our study has several limitations. We did not isolate viruses, and the test for virus neutralization was not conducted. We did not investigate whether the presence of cattle in small ruminant flocks is a risk factor for pestiviral infection. Also, whole-genome sequencing of the detected BVDV strains was not performed; only partial 5′ UTR sequencing was conducted for 14 BVDV strains.

## 5. Conclusions

Pestiviral diseases of livestock impose significant economic costs through decreased productivity, direct veterinary expenses, and the implementation of control measures. We present the first nationwide cross-sectional survey of prevalence for pestiviruses in sheep and goats, with samples collected from 2514 animals across 148 herds in all 17 oblasts of Kazakhstan from November 2022 to December 2024. The study found that 53.7% of individual sheep and goats (1088 out of 2028) and 86.7% of herds (78 out of 90) were positive for BVDV/BDV antibodies. These findings offer valuable insight into the epidemiology of pestiviruses in small ruminants, highlighting the importance of integrated surveillance and control measures to mitigate the broader impact of pestiviral diseases on diverse ruminant populations and ecosystems. We also identified risk factors associated with pestiviral infection in sheep and goats in Kazakhstan. The constructed risk map for the spread of pestiviruses at the district level, in conjunction with data on the established prevalence of ruminant pestiviruses, clearly highlights which specific districts require focused attention for preventive control measures.

A phylogenetic analysis of Kazakhstani 14 BVDV2 partial 5′UTR genomes led to their clustering into two major groups with a low mean nucleotide intragroup divergence reaching 1%. Both groups of Kazakhstani BVDV2 strains could potentially be classified as separate BVDV2 genotypes according to the results of BLAST and phylogenetic analyses.

The results indicate that pestiviruses from sheep and goats should be considered a significant source of infection for domestic cattle. This situation may disrupt serological and molecular surveillance for BVD. Therefore, sheep and goats should be included in vaccination and eradication programs.

## Figures and Tables

**Table 1:** Primers and probes used in the study.

Primer/Probe Name	Primer Sequence (5′ → 3′)	Orientation	Pathogen	Genome Position	T_a_ [°C]	Ref.
PestiV-189-qF	5′-GHAGTCGTCARTGGTTCGAC	F	BVDV1 BVDV2 BDV HoBiPeV	188–207 ^1^ 189–208 ^2^ 177–196 ^3^ 184–202 ^4^	57	[38], modified
PestiV-396-qR	5′-WCAACTCCATGTGCCATGTAC	R	BVDV1 BVDV2 BDV HoBiPeV	375–395 ^1^ 376–396 ^2^ 362–382 ^3^ 373–393 ^4^	57	[39], modified
PestiV-235-qPr	FAM-5′-TGCCAYGTGGACGAGGGCATGC-BHQ1	F	BVDV1 BVDV2 BDV HoBiPeV	231–252 ^1^ 235–256 ^2^ 220–241 ^3^ 225–246 ^4^	57	[40]
BVDV-101-F	5′-GCTAGCCATGCCCTTAGTAG	F	BVDV1 BVDV2	101–120 ^1^ 102–121 ^2^	58	[41]
BVDV-105-nF	5′-GCCATGCCCTTAGTAGGACTAGC	F	BVDV1 BVDV2	105–127 ^1^ 106–128 ^2^	58	[42]
PestiV-394-R	5′-CAACTCCATGTGCCATGTACAGC	R	BVDV1 BVDV2 BDV HoBiPeV	372–394 ^1^ 373–395 ^2^ 359–381 ^3^ 370–392 ^4^	58	[42]
BDV-136-F	5′-TCGTGGTGAGATCCCTGAG	F	BDV	136–153 ^3^	58	[43]
BDV-360-R	5′-GCAGAGATTTTTTATACTAGCCTATDC	R	BDV	335–360 ^3^	56	[43]
HoBi-134-F	5′-GACTAGTGGTGGCAGTGAGC	F	HoBiPeV	134–153 ^4^	56	[44]
HoBi-326-nR	5′-ATCAGGCTGTACTCCCAAAG	R	HoBiPeV	307–326 ^4^	56	[45]

^1^ Primer and probe positions refer to the sequences of the reference BVDV1 genome [46] (GenBank: M31182). ^2^ Primer and probe positions refer to the sequences of reference BVDV2 genome [47] (GenBank: LC006970). ^3^ Primer and probe positions refer to the sequences of the reference BDV genome [48] (GenBank: FJ040215). ^4^ Primer and probe positions refer to the sequences of the reference BDV genome [49] (GenBank reference sequence: NC_003679). Abbreviations: F—forward; R—reverse; Ref—reference; T_a_—annealing temperature.

**Table 2:** Apparent animal-level prevalence of BVDV/BDV antibodies and pestiviral RNA in sheep in Kazakhstan, by oblast.

Region/ Oblast	Sampled (Herds Animals)	Antibody ELISA			RT-qPCR			No. of Conventional RT-PCR-Positive/No. of Confirmed by Sanger Sequencing		
		No. Tested (Herds/ Animals)	No. of Positive Herds (%)	No. of Positive Animals (%)	No. Tested (Herds/ Animals	No. of Positive Herds (%)	No. of Positive Animals (%)	BVDV	BDV	HoBiPeV
Southern Kazakhstan										
Zhambyl	3/120	3/92	3 (100.0)	81 (88.0)	3/92	3 (100.0)	7 (7.6)	7/4	0	0
Turkistan	6/120	6/120	6 (100.0)	79 (65.8)	5/92	3 (60.0)	15 (16.3)	13/6	0	0
Almaty	5/120	5/118	3 (60.0)	47 (39.8)	5/118	1 (20.0)	1 (0.9)	1/0	0	0
Jetisu	5/115	5/110	5 (100.0)	86 (78.2)	5/94	2 (40.0)	2 (2.1)	2/1	0	0
Kyzylorda	5/120	5/118	3 (60.0)	49 (41.5)	5/120	2 (40.0)	3 (2.5)	3/1	0	0
Western Kazakhstan										
Mangystau	16/283	5/228	5 (100.0)	41 (18.0)	16/182	3 (18.8)	5 (2.8)	4/2	0	0
West Kazakhstan	8/120	8/118	8 (100.0)	91 (77.1)	8/120	2 (25.0)	2 (1.7)	1/0	0	0
Atyrau	6/120	6/118	6 (100.0)	19 (16.1)	6/118	0	0	-	-	-
Aktobe	5/120	4/94	4 (80.0)	79 (84.0)	5/120	1 (20.0)	2 (1.7)	2/2	0	0
Central Kazakhstan										
Karagandy	20/309	8/104	6 (75.0)	53 (51.0)	12/180	0	0	-	-	-
Ulytau	6/125	6/124	3 (50.0)	48 (38.7)	5/124	0	0	-	-	-
Eastern Kazakhstan										
East Kazakhstan	6/145	4/48	4 (100.0)	36 (75.0)	6/139	2 (33.3)	4 (2.9)	4/1	0	0
Abai	39/170	8/118	7 (87.5)	39 (33.1)	39/168	8 (20.5)	11 (6.6)	10/5	0	0
Northern Kazakhstan										
Kostanai	3/102	3/102	3 (100.0)	59 (57.8)	3/94	0	0	-	-	-
North Kazakhstan	7/245	7/245	7 (100.0)	189 (77.1)	7/120	0	0	-	-	-
Akmola	6/130	5/125	3 (60.0)	60 (48.0)	5/125	0	0	-	-	-
Pavlodar	2/50	2/46	2 (100.0)	32 (69.6)	2/50	0	0	-	-	-
TOTAL	148/2514	90/2028	78 (86.7)	1088 (53.7)	137/2056	27 (19.7)	52 (2.5)	47/22	0	0

**Table 3:** Univariate analysis for the association between animal factors and apparent animal-level BVDV/BDV seropositivity in sheep and goats in Kazakhstan (n = 2028).

Variable/ Category	No. Tested	ELISA Positive (%)	OR (95% CI)	Heterogeneity			Logistic Regression			
				χ^2^	*p*-Value	A/B/C	OR (95% CI)	*b*	S.E.	*p*-Value
Age										
≥2 years	1056	613 (58.1)	1.5 (1.2–1.7)	17.16	<0.0001 *	B	1.6 (1.3–2.1)	0.48	0.13	0.0001 *
[1–2) years	626	315 (50.3)	0.8 (0.7–0.9)	4.03	0.0446 *	A	1.2 (0.9–1.5)	0.16	0.13	0.2236
<1 years	346	160 (46.2)	0.7 (0.6–0.9)	9.20	0.0024 *	A	Ref	Ref	Ref	Ref
Sex										
Female	1668	902 (54.1)	1.1 (0.9–1.4)	0.41	<0.4057	A	1.1 (0.9–1.4)	0.10	0.12	0.4049
Male	360	186 (51.7)				A	Ref	Ref	Ref	Ref
Species										
Sheep	1980	1068 (53.9)	1.6 (0.9–2.9)	2.84	0.0921	A	1.6 (0.9–2.9)	0.49	0.30	0.0957
Goats	48	20 (41.7)				A	Ref	Ref	Ref	Ref
Property form										
Personal subsidiary households	1007	369 (36.6)	0.2 (0.2–0.3)	232.61	<0.0001 *	A	0.9 (0.7–1.1)	−0.13	0.14	0.3221
Peasant farms	722	600 (83.1)	8.2 (6.6–10.3)	391.1	<0.0001 *	B	7.4 (5.5–10.0)	2.0	0.15	<0.0001 *
Limited Liability Companies and State Enterprises	299	119 (39.8)	0.5 (0.4–0.7)	27.05	<0.0001 *	A	Ref	Ref	Ref	Ref
Herd size										
≥500 heads	304	209 (68.8)	2.1 (1.6–2.7)	32.80	<0.0001 *	A	2.6 (2.0–3.5)	0.96	0.15	<0.0001 *
[100–500) heads	1069	579 (54.2)	1.0 (0.8–1.2)	0.24	0.6243	B	1.4 (1.2–1.7)	0.340	0.10	0.0008
<100 heads	655	300 (45.8)	0.6 (0.5–0.8)	23.95	<0.0001 *	C	Ref	Ref	Ref	Ref
Region										
Northern Kazakhstan	518	340 (65.6)	1.9 (1.6–2.4)	40.19	<0.0001 *	A	2.3 (1.6–3.3)	0.84	0.18	<0.0001 *
Western Kazakhstan	558	230 (41.2)	0.5 (0.4–0.6)	47.81	<0.0001 *	B	0.9 (0.6–1.2)	−0.16	0.18	0.3643
Southern Kazakhstan	558	342 (61.3)	1.5 (1.3–1.9)	18.07	<0.0001 *	A	1.9 (1.4–2.4)	0.65	0.18	0.0003 *
Central Kazakhstan	228	101 (44.3)	0.6 (0.5–0.9)	9.03	0.0027 *	B	1.0 (0.6–1.4)	−0.04	0.21	0.8612
Eastern Kazakhstan	166	75 (45.2)	0.7 (0.5–0.9)	5.23	0.0225 *	B	Ref	Ref	Ref	Ref

χ^2^: Pearson’s chi-square test; OR: odds ratio; CI 95%: 95% confidence interval of OR; *b*: regression coefficient; S.E.: standard error of *b*; Ref: reference group. * Significant values (*p* < 0.05); A/B/C: values represented by the same letter in the column do not show any significant difference (*p* > 0.05).

**Table 4:** Multivariate logistic analysis for the association between animal risk factors and pestivirus seropositivity in sheep and goats in Kazakhstan (n = 2028).

Risk Factor	OR	95% CI	*b*	S.E.	Z	*p*-Value
Age of test subjects over 2 years	1.71	1.40–2.09	0.54	0.10	5.27	<0.0001 *
(Reference group: age under 2 years)						
Peasant farms	7.52	5.94–9.52	2.02	0.12	16.77	<0.0001 *
(Reference group: all other property forms)						
Herd size of more than 500 heads	1.61	1.20–2.17	0.48	0.15	3.18	0.0016
(Reference group: herds fewer than 500 heads)						
Northern or southern region of Kazakhstan	1.30	1.06–1.60	0.26	0.11	2.49	0.0129 *
(Reference group: all other regions)						
Constant	-	-	−0.97	0.09	−10.40	<0.0001 *
Score: 429.0351; *p* < 0.0001						
Likelihood Ratio: 463.9471; *p* < 0.0001						

OR: odds ratio; CI 95%: 95% confidence interval of OR; *b*: regression coefficient; S.E.: standard error of *b*; Z: linear predictor. * Significant values (*p* < 0.05).

**Table 5:** Univariate analysis for the association between economic and geographic risk factors and pestivirus seroprevalence in sheep and goats in Kazakhstan (n = 2028).

Risk Factor/Category	No. Tested	No. ELISA Positive (%)	χ^2^	Logistic Regression			
				OR (95% CI)	*b*	S.E.	*p*-Value
Density of small ruminants							
≥10 heads/sq.km	562	373 (66.4)	50.62	2.1 (1.7–2.5)	0.73	0.10	<0.0001 *
<10 heads/sq.km	1466	715 (48.8)		Ref	Ref	Ref	Ref
Density of cattle							
≥5 heads/sq.km	675	443 (65.6)	58.40	2.1 (1.7–2.5)	0.74	0.10	<0.0001 *
<5 heads/sq.km	1353	645 (47.7)		Ref	Ref	Ref	Ref
Density of wild ruminants							
≥5 heads/100 sq.km	1316	665 (50.5)	14.65	0.7 (0.6–0.8)	−0.36	0.09	0.0001 *
<5 heads/100 sq.km	712	423 (59.4)		Ref	Ref	Ref	Ref
Livestock importation in 2022–2024 (>50 heads)							
Yes	636	394 (62.0)	25.66	1.6 (1.4–2.0)	0.49	0.10	<0.0001 *
No	1392	694 (49.9)		Ref	Ref	Ref	Ref
Density of automobile routes							
≥100 m/sq.km	588	409 (69.6)	84.28	2.6 (2.1–3.1)	0.94	0.10	<0.0001 *
<100 m/sq.km	1440	679 (47.2)		Ref	Ref	Ref	Ref
Share of sheep and goat population in backyard farms							
≥50%	1296	753 (58.1)	28.63	1.6 (1.4–1.9)	0.50	0.09	<0.0001 *
<50%	732	335 (45.8)		Ref	Ref	Ref	Ref
Shared border with countries where pestiviral infections are endemic							
Yes	434	314 (72.4)		2.8 (2.2–3.5)	1.02	0.12	<0.0001 *
No	1594	774 (48.6)		Ref	Ref	Ref	Ref
Proximity to the sites of previous BVD outbreaks							
Within the 100 km protection zone	683	365 (53.4)	0.02	1.0 (0.8–1.2)	−0.01	0.09	0.8934
Outside the 100 km protection zone	1345	723 (53.8)		Ref	Ref	Ref	Ref

χ^2^: Pearson’s chi-square test; OR: Odds ratio; Ref: reference group. 95% CI: 95% confidence interval; * Significant values (*p* < 0.05) in the logistic regression.

**Table 6:** Multivariate logistic analysis of the association between economic and geographic risk factors and pestivirus seropositivity in sheep and goats in Kazakhstan (n = 2028).

Risk Factor	OR	95% CI	*b*	S.E.	Z	*p*-Value
High density of small ruminants	0.98	0.71–1.36	−0.02	0.17	−0.12	0.9056
Density of cattle	1.33	0.99–1.78	0.29	0.15	1.93	0.0542
Livestock importation	1.55	1.23–1.90	0.44	0.10	4.28	<0.0001 *
High proportion of backyard husbandry	1.45	1.20–1.77	0.37	0.10	3.79	0.0002 *
High road density	2.10	1.65–2.68	0.74	0.12	6.02	<0.0001 *
High density of wild ruminants	0.78	0.64–0.95	−0.25	0.10	−2.43	0.0153 *
Shared border with countries where BVD is endemic	2.39	1.87–3.05	0.87	0.13	6.96	<0.0001 *
Constant	-	-	−0.54	0.12	−4.62	<0.0001 *
Score: 200.5718; *p* < 0.0001						
Likelihood Ratio: 208.6671; *p* < 0.0001						

OR: odds ratio; 95% CI: 95% confidence interval of OR; *b*: regression coefficient; S.E.: standard error of *b*; Z: linear predictor. * Significant values (*p* < 0.05).

## Data Availability

The nucleotide sequences reported in this paper are deposited into the NCBI GenBank database [50] under the accession numbers listed in the text.

## Supplementary Materials

The following supporting information can be downloaded at: https://www.mdpi.com/article/10.3390/v17050676/s1. Table S1: Data regarding the populations of cattle, sheep, and goats, along with the vaccination plan against BVDV, categorized by oblasts of Kazakhstan.

## Abbreviations

The following abbreviations are used in this manuscript:

BLAST	Basic GenBank Local Alignment Search Tool
BDV	Border disease virus
BVD	Bovine viral diarrhea
BVDV	Bovine viral diarrhea virus
95% CI	95% Confidence interval
HoBiPeV	HoBi-like pestivirus
MEGA	Molecular Evolutionary Genetics Analysis
OR	Odds ratio
PI	Persistent infection
5′UTR	5′-untranslated region

## References

[1] Bisschop P.I.H., Strous E.E.C., Waldeck H.W.F., van Duijn L., Mars M.H., Santman-Berends I.M.G.A., Wever P., van Schaik G. (2025). Risk factors for the introduction of bovine viral diarrhea virus in the context of a mandatory control program in Dutch dairy herds. J. Dairy Sci..

[2] Nelson D.D., Duprau J.L., Wolff P.L., Evermann J.F. (2016). Persistent bovine viral diarrhea virus infection in domestic and wild small ruminants and camelids including the mountain goat (*Oreamnos americanus*). Front. Microbiol..

[3] Evans C.A., Reichel M.P. (2021). Non-bovine species and the risk to effective control of bovine viral diarrhoea (BVD) in cattle. Pathogens.

[4] Fray M.D., Paton D.J., Alenius S. (2000). The effects of bovine viral diarrhoea virus on cattle reproduction in relation to disease control. Anim. Reprod. Sci..

[5] Baker J.C. (1995). The clinical manifestations of bovine viral diarrhea infection. Vet. Clin. N. Am. Food Anim. Pract..

[6] Grooms D.L. (2004). Reproductive consequences of infection with bovine viral diarrhea virus. Vet. Clin. N. Am. Food Anim. Pract..

[7] Toplak I., Hostnik P., Černe D., Mrkun J., Starič J. (2021). The principles of the voluntary programme for the control and elimination of bovine viral diarrhoea virus (BVDV) from infected herds in Slovenia. Front. Vet. Sci..

[8] Walz P.H., Grooms D.L., Passler T., Ridpath J.F., Tremblay R., Step D.L., Callan R.J., Givens M.D., American College of Veterinary Internal Medicine (2010). Control of bovine viral diarrhea virus in ruminants. J. Vet. Intern. Med..

[9] Diao N.C., Chen Z.Y., Shi J.F., Wang Q., Sheng C.Y., Ma B.Y., Yang Y., Sun Y.H., Shi K., Du R. (2021). Prevalence of Bovine Viral Diarrhea Virus in Ovine and Caprine Flocks: A global systematic review and meta-analysis. Front. Vet. Sci..

[10] Postel A., Smith D.B., Becher P. (2021). Proposed update to the taxonomy of Pestiviruses: Eight additional species within the genus *Pestivirus*, family *Flaviviridae*. Viruses.

[11] Deng M., Ji S., Fei W., Raza S., He C., Chen Y., Chen H., Guo A. (2015). Prevalence study and genetic typing of bovine viral diarrhea virus (BVDV) in four bovine species in China. PLoS ONE.

[12] Yeşilbağ K., Alpay G., Becher P. (2017). Variability and global distribution of subgenotypes of Bovine Viral Diarrhea Virus. Viruses.

[13] Malacari D.A., Pecora A., Perez Aguirreburualde M.S., Cardoso N.P., Odeon A.C., Capozzo A.V. (2018). In vitro and in vivo characterization of a typical and a high pathogenic bovine viral diarrhea virus type II strains. Front. Vet. Sci..

[14] Zhu J., Wang C., Zhang L., Zhu T., Li H., Wang Y., Xue K., Qi M., Peng Q., Chen Y. (2022). Isolation of BVDV-1a, 1m, and 1v strains from diarrheal calf in china and identification of its genome sequence and cattle virulence. Front. Vet. Sci..

[15] Ridpath J.F. (2005). Practical significance of heterogeneity among bvdv strains: Impact of biotype and genotype on U.S. control programs. Prev. Vet. Med..

[16] Alpay G., Yesilbag K. (2015). Serological relationships among subgroups in bovine viral diarrhea virus genotype 1 (BVDV-1). Vet. Microbiol..

[17] Fulton R.W., Cook B.J., Payton M.E., Burge L.J., Step D.L. (2020). Immune response to bovine viral diarrhea virus (BVDV) vaccines detecting antibodies to BVDV subtypes 1a, 1b, 2a, and 2c. Vaccine.

[18] Kaiser V., Nebel L., Schüpbach-Regula G., Zanoni R.G., Schweizer M. (2017). Influence of border disease virus (BDV) on serological surveillance within the bovine virus diarrhea (BVD) eradication program in Switzerland. BMC Vet. Res..

[19] Muasya D., Leeuwen J.V., Gitau G., McKenna S., Heider L., Muraya J. (2022). Evaluation of antibody and antigen cross-reaction in Kenyan dairy cattle naturally infected with two pestiviruses: Bovine viral diarrhea virus and classical swine fever virus. Vet. World.

[20] Oguzoglu T.C., Tan M.T., Toplu N., Demir A.B., Bilge-Dagalp S., Karaoglu T., Ozkul A., Alkan F., Burgu I., Haas L. (2009). Border disease virus (BDV) infections of small ruminants in Turkey: A new BDV subgroup?. Vet. Microbiol..

[21] Li W., Mao L., Zhao Y., Sun Y., He K., Jiang J. (2013). Detection of border disease virus (BDV) in goat herds suffering diarrhea in eastern China. Virol. J..

[22] Righi C., Petrini S., Pierini I., Giammarioli M., De Mia G.M. (2021). Global Distribution and Genetic Heterogeneity of Border Disease Virus. Viruses.

[23] Shi H., Kan Y., Yao L., Leng C., Tang Q., Ji J., Sun S. (2016). Identification of Natural Infections in Sheep/Goats with HoBi-like Pestiviruses in China. Transbound. Emerg. Dis..

[24] Decaro N. (2020). HoBi-like pestivirus and reproductive disorders. Front. Vet. Sci..

[25] Nagai M., Hayashi M., Sugita S., Sakoda Y., Mori M., Murakami T., Ozawa T., Yamada N., Akashi H. (2004). Phylogenetic analysis of bovine viral diarrhea viruses using five different genetic regions. Virus Res..

[26] de Oliveira P.S.B., Silva Júnior J.V.J., Weiblen R., Flores E.F. (2021). Subtyping bovine viral diarrhea virus (BVDV): Which viral gene to choose?. Infect. Genet. Evol..

[27] Statistical Agency of the Republic of Kazakhstan Dynamics of the Main Socio-Economic Indicators in the Regions of Kazakhstan. http://www.stat.gov.kz/region.

[28] CABI Compendium Bovine Viral Diarrhea. http://www.stat.gov.kz/region.

[29] Official Website of the State of the Republican Veterinary Laboratory, Ministry of Agriculture of the Republic of Kazakhstan. https://rvl.kz/ru/.

[30] State Register of Veterinary Drugs and Feed Additives Official Website of the Ministry of Agriculture of the Republic of Kazakhstan. https://www.gov.kz/memleket/entities/moa/documents/details/471966?lang=en.

[31] Zhigailov A.V., Perfilyeva Y.V., Ostapchuk Y.O., Kan S.A., Lushova A.V., Kuligin A.V., Ivanova K.R., Kuatbekova S.A., Abdolla N., Naizabayeva D.A. (2023). Molecular and serological survey of bovine viral diarrhea virus infection in cattle in Kazakhstan. Res. Vet. Sci..

[32] Zhigailov A.V., Perfilyeva Y.V., Ostapchuk Y.O., Kulemin M.V., Ivanova K.R., Abdolla N., Kan S.A., Maltseva E.R., Berdygulova Z.A., Naizabayeva D.A. (2023). Molecular detection and characterization of bovine viral diarrhea virus type 2 and bluetongue virus 9 in forest flies (*Hippobosca equina*) collected from livestock in southern Kazakhstan. Vet. Parasitol. Reg. Stud. Rep..

[33] CABI Compendium Border Disease. http://www.stat.gov.kz/region.

[34] Charan J., Kantharia N.D. (2013). How to calculate sample size in animal studies?. J. Pharmacol. Pharmacother..

[35] Dohoo I., Martin W., Stryhn H. (2009). Veterinary Epidemiologic Research.

[36] Otte M.J., Gumm I. (1997). Intra-cluster correlation coefficients of 20 infections calculated from the results of cluster-sample surveys. Prev. Vet. Med..

[37] Chatterjee S., Premachandran S., Bagewadikar R.S., Poduval T.B. (2005). The use of ELISA to monitor amplified hemolysis by the combined action of osmotic stress and radiation: Potential applications. Radiat. Res..

[38] Hoffmann B., Depner K., Schirrmeier H., Beer M. (2006). A universal heterologous internal control system for duplex real-time RT-PCR assays used in a detection system for pestiviruses. J. Virol. Methods.

[39] Vilcek S., Herring A.J., Herring J.A., Nettleton P.F., Lowings J.P., Paton D.J. (1994). Pestiviruses isolated from pigs, cattle and sheep can be allocated into at least three genogroups using polymerase chain reaction and restriction endonuclease analysis. Arch. Virol..

[40] Gaede W., Reiting R., Schirrmeier H., Depner K.R., Beer M. (2005). Detection and species-specific differentiation of pestiviruses using real-time RT-PCR. Berl. Munch. Tierarztl. Wochenschr..

[41] Yang N., Xu M., Ma Z., Li H., Song S., Gu X., Liu J., Yang Z., Zhu H., Ma H. (2023). Detection of emerging HoBi-like Pestivirus (BVD-3) during an epidemiological investigation of bovine viral diarrhea virus in Xinjiang: A first-of-its-kind report. Front. Microbiol..

[42] Young N.J., Thomas C.J., Collins M.E., Brownlie J. (2006). Real-time RT-PCR detection of Bovine Viral Diarrhoea virus in whole blood using an external RNA reference. J. Virol. Methods.

[43] Vilbek S., Paton D.J. (2000). A RT-PCR assay for the rapid recognition of border disease virus. Vet. Res..

[44] Liu L., Xia H., Bela’k S., Baule C. (2008). A TaqMan real-time RT-PCR assay for selective detection of atypical bovine pestiviruses in clinical samples and biological products. J. Virol. Methods.

[45] Mari V., Losurdo M., Lucente M.S., Lorusso E., Elia G., Martella V., Patruno G., Buonavoglia D., Decaro N. (2016). Multiplex real-time RT-PCR assay for bovine viral diarrhea virus type 1, type 2 and HoBi-like pestivirus. J. Virol. Methods.

[46] Colett M.S., Larson R., Gold C., Strick D., Anderson D.K., Purchio A.F. (1988). Molecular cloning and nucleotide sequence of the pestivirus bovine viral diarrhea virus. Virology.

[47] Sato A., Kameyama K., Nagai M., Tateishi K., Ohmori K., Todaka R., Katayama K., Mizutani T., Yamakawa M., Shirai J. (2015). Complete Genome Sequence of Bovine Viral Diarrhea Virus 2 Japanese Reference and Vaccine Strain KZ-91CP. Genome Announc..

[48] Liu L., Kampa J., Belák S., Baule C. (2009). Virus recovery and full-length sequence analysis of atypical bovine pestivirus Th/04_KhonKaen. Vet. Microbiol..

[49] Becher P., Orlich M., Thiel H.J. (1998). Complete genomic sequence of border disease virus, a pestivirus from sheep. J. Virol..

[50] National Center for Biotechnology Information (NCBI). https://www.ncbi.nlm.nih.gov.

[51] Kumar S., Stecher G., Li M., Knyaz C., Tamura K. (2018). MEGA X: Molecular Evolutionary Genetics Analysis across computing platforms. Mol. Biol. Evol..

[52] Felsenstein J. (1985). Confidence limits on phylogenies: An approach using bootstrap. Evolution.

[53] Afanasiev A.V., Bazhanov V.S., Korelov M.N., Sludsky A.A., Strauman E.I. (1953). Animals of Kazakhstan.

[54] Statistical Agency of the Republic of Kazakhstan Statistics of Agriculture, Forestry, Hunting, and Fisheries. https://stat.gov.kz/en/industries/business-statistics/stat-forrest-village-hunt-fish.

[55] Meijer J.R., Huijbregts M.A.J., Schotten K.C.G.J., Schipper A.M. (2018). Global patterns of current and future road infrastructure. Environ. Res. Lett..

[56] Official Website of the State Inspection Committee in the Agro-Industrial Complex of the Ministry of Agriculture of the Republic of Kazakhstan. https://www.gov.kz/memleket/entities/agroindust?lang=en.

[57] Kimura M. (1980). A simple method for estimating evolutionary rates of base substitutions through comparative studies of nucleotide sequences. J. Mol. Evol..

[58] Oem J.K., Lee E.Y., Byun J.W., Byun J.-W., Kim H.-Y., Kwak D.-M., Song H.-J., Jung B.-Y. (2012). Serological and virological investigation of pestiviruses in Korean black goat. Korean J. Vet. Serv..

[59] Kalaiyarasu S., Mishra N., Rajukumar K., Nema R.K., Behera S.P. (2015). Development and evaluation of a truncated recombinant NS3 antigen-based indirect ELISA for detection of Pestivirus antibodies in sheep and goats. J. Immunoass. Immunochem..

[60] Danuser R., Vogt H.R., Kaufmann T., Peterhans E., Zanoni R. (2009). Seroprevalence and characterization of pestivirus infections in small ruminants and new world camelids in Switzerland. Schweiz Arch Tierheilkd.

[61] Su N., Wang Q., Liu H.Y., Li L.M., Tian T., Yin J.Y., Zheng W., Ma Q.X., Wang T.T., Li T. (2023). Prevalence of bovine viral diarrhea virus in cattle between 2010 and 2021: A global systematic review and meta-analysis. Front. Vet. Sci..

[62] Ma J.G., Cong W., Zhang F.H., Feng S.Y., Zhou D.H., Wang Y.M., Zhu X.Q., Yin H., Hu G.X. (2016). Seroprevalence and risk factors of bovine viral diarrhoea virus (BVDV) infection in yaks (*Bos grunniens*) in northwest China. Trop. Anim. Health Prod..

[63] Deng M., Chen N., Guidarini C., Xu Z., Zhang J., Cai L., Yuan S., Sun Y., Metcalfe L. (2020). Prevalence and genetic diversity of bovine viral diarrhea virus in dairy herds of China. Vet. Microbiol..

[64] Xiao Y., Liu Y., Chi T., Jiang W., He T., Xu L., Dong Q., Chen R.Q., An Z., Sun X. (2025). Prevalence and genetic characterization of bovine viral diarrhea virus in dairy cattle in northern China. BMC Vet. Res..

[65] Glotov A.G., Glotova T.I., Nefedchenko A.V., Koteneva S.V. (2022). Genetic diversity and distribution of bovine pestiviruses (*Flaviviridae*: *Pestivirus*) in the world and in the Russian Federation. Vopr. Virusol..

[66] Manandhar S., Yadav G.P., Singh D.K. (2018). Epidemiological survey of bovine viral diarrhea in dairy cattle in Nepal. OIE Bullet. Newsfeed.

[67] Sayers R.G., Byrne N., O’Doherty E., Arkins S. (2015). Prevalence of exposure to bovine viral diarrhoea virus (BVDV) and bovine herpesvirus-1 (BoHV-1) in Irish dairy herds. Res. Vet. Sci..

[68] Rüfenacht J., Schaller P., Audigé L., Peterhans E. (2012). Prevalence of cattle infected with bovine viral diarrhoea virus in Switzerland. Vet. Rec..

[69] Ince O.B. (2022). The seroepidemiology of pestivirus infection in sheep in Afyonkarahisar province of Turkey and the analysis of associated risk factors. J. Hell. Vet. Med. Soc..

[70] van Roon A.M., Mercat M., van Schaik G., Nielen M., Graham D.A., More S.J., Guelbenzu-Gonzalo M., Fourichon C., Madouasse A., Santman-Berends I.M.G.A. (2020). Quantification of risk factors for bovine viral diarrhea virus in cattle herds: A systematic search and meta-analysis of observational studies. J. Dairy Sci..

[71] Tura T., Tamiru Y., Dima C., Garoma A., Kebede A., Abdeta D. (2025). Seroprevalence of bovine viral diarrhea virus infection and its associated risk factors in dairy cattle in and around Sebeta sub city, Ethiopia. Sci. Rep..

[72] Moennig V., Yarnall M.J. (2021). The Long Journey to BVD Eradication. Pathogens.

