# Supplementary material for: Survey and Associated Risk Factors for the Presence of Ruminant Pestiviruses in Domestic Ovine and Caprine Populations from Kazakhstan
Source: Viruses. 2025 May 6;17(5):676. doi: 10.3390/v17050676 (PMC12115794; doi:10.3390/v17050676)
Supplement: Supplementary file 1 [file viruses-17-00676-s001.zip › viruses-3588775-supplementary.pdf]

Supplementary Table S1. Data regarding the populations of cattle, sheep, and goats, along with the vaccination plan against BVDV, categorized by oblasts of Kazakhstan

Region / Oblast	Sheep population [1]	Goat population [1]	Cattle population [1]	State vaccination plan against BVDV [2]	The coverage of cattle heads by the state vaccination program, %
Southern Kazakhstan					
Zhambyl	3,011,084	92,194	429,090	-	0.0
Turkistan	4,283,779	183,760	1,167,884	-	0.0
Almaty	2,196,542	116,045	581,007	12,910	2.2
Jetisu	1,377,662	180,389	499,638	6000	1.2
Kyzylorda	525,628	120,632	362,887	-	0.0
Western Kazakhstan					
Mangystau	227,422	58,304	19,688	-	0.0
West Kazakhstan	1,190,400	172,371	927,595	-	0.0
Atyrau	450,897	113,232	225,510	3500	1.6
Aktobe	1,070,340	126,033	596,440	15,230	2.6
Central Kazakhstan					
Karagandy	559,089	127,272	446,817	20,500	4.6
Ulytau	199,065	43,579	149,250	11,000	7.4
Eastern Kazakhstan					
East Kazakhstan	456,629	88,451	361,447	-	0.0
Abai	1,030,840	107,834	708,126	-	0.0
Northern Kazakhstan					
Kostanai	400,394	43,938	398,299	21,150	5.3
North Kazakhstan	378,865	8527	288,846	27,200	9.4
Akmola	576,568	34,586	393,183	13,150	3.3
Pavlodar	593,132	59,207	483,720	8000	1.7
TOTAL	20,210,671	1,682,335	8,039,427	138,640	1.7

[1] As per 1 February 2025 [27]. [2] Data for 2024 is presented [29].
